# Strengthening laboratory networks in the Central Africa region: A milestone for epidemic preparedness and response

**DOI:** 10.4102/ajlm.v11i1.1492

**Published:** 2022-05-19

**Authors:** Patrick A. Njukeng, Charles Njumkeng, Callistus Ntongowa, Mohammed Abdulaziz

**Affiliations:** 1 Global Health Systems Solutions, Douala, Cameroon; 2 Africa Centres for Disease Control and Prevention, Addis Ababa, Ethiopia

**Keywords:** COVID 19, laboratory, health system, strengthening, network, quality, management, response

## Abstract

**Background:**

Health systems in the Central Africa region are among the weakest and least funded in the world. The lack of laboratory networks and adequately trained personnel with clearly defined responsibilities has hampered the implementation of laboratory quality improvement programmes. Global Health Systems Solutions (GHSS) obtained a grant from the Africa Centres for Disease Control and Prevention to develop laboratory networks for disease surveillance and strengthen the quality of laboratory testing in the Central Africa region.

**Intervention:**

One year after the grant was awarded on 01 October 2018, GHSS has launched a Regional Integrated Surveillance and Laboratory Network (RISLNET) for Central Africa and developed National Laboratory Strategic Plans and Policies for member states, eight frameworks and guideline documents, as well as a website for RISLNET Central Africa. GHSS has also launched an Extension for Community Health Outcomes platform to supervise laboratories enrolled for accreditation, installed a Basic Laboratory Information System (BLIS) in four laboratories in four member states, and trained 247 laboratory personnel and laboratory experts on BLIS, quality assurance, external quality assurance, Strengthening Laboratory Management Towards Accreditation (SLMTA), quality management systems, and equipment maintenance and calibration.

**Lessons learnt:**

Participating laboratories now serve as reference laboratories for COVID-19 testing in various countries. Point-of-care testing, using the GeneXpert platform, has been the central strategy for the scale-up of COVID-19 testing in the Central Africa region.

**Recommendations:**

Expanding SLMTA to other laboratories within Central Africa will significantly improve the quality management of laboratories for a better healthcare system.

## Background

Clinical laboratories form an essential component of the health system. In addition to providing test results for disease diagnosis and guiding treatment by detecting drug resistance, they form the backbone of disease surveillance and public health response to outbreaks and epidemics.^1,2,3^ Efforts to strengthen health systems should therefore focus on laboratory services and systems as they provide primary information that informs decision making for best healthcare outcomes.^4^

The role of the laboratory in responding to epidemics is undoubtedly a vital one judging from the lessons learned in recent years. The 2014 Ebola outbreak in West Africa revealed the need to organise and maintain laboratory systems or networks that can rapidly and effectively adjust to carry out new diagnostic testing or laboratory services in response to large-scale epidemics.^5^ Another lesson learned from the Ebola outbreak was the need to set up a platform that enables planning and preparedness activities to be rapidly adapted to emerging pathogens or outbreaks.^6^ The current coronavirus disease 2019 (COVID-19) pandemic has further emphasised the overarching role of quality laboratory systems in disease outbreak preparedness and response in Africa and, specifically, in the Central Africa region.

Health systems in the Central Africa region are among the weakest in the world and have received the least funding support when compared to other regions.^7,8^ However, funding sources and advanced testing technologies are increasingly being made available to laboratories in the region from bodies such as the African Union/Africa Centres for Disease Control and Prevention (CDC), African Society for Laboratory Medicine, the World Health Organization’s Regional Office for Africa and the United States CDC, among others. Nonetheless, laboratory quality improvement is still hindered by the lack of adequately trained personnel with clearly defined responsibilities, as well as the lack of well-established organisations and laboratory networks. To promote the strengthening of laboratory systems, the network of national public health institutes needs to be strengthened through continuous advocacy and staff capacity development.

Global Health Systems Solutions (GHSS), an international non-governmental organisation based in Cameroon, obtained a grant from Africa CDC to establish and strengthen public health laboratory systems and networks in the Central Africa region. GHSS worked in close collaboration with Africa CDC and the Regional Collaboration Center to achieve this strategic vision of the Africa CDC.

The principal goal of the African Union/Africa CDC grant was to develop laboratory networks for disease surveillance and strengthen the quality of laboratory testing across nine countries in the Central Africa region, namely Gabon, Cameroon, Central African Republic, Chad, Congo Brazzaville, Equatorial Guinea, Burundi, Democratic Republic of Congo, and Sao Tome and Principe. This goal was predicated on three key objectives. The first objective was to develop a framework and statute for the Central Africa Regional Integrated Surveillance and Laboratory Network (RISLNET) that defines its function and operations, permitting the establishment of a website to share framework documents and experiences between the different countries. The second objective was to implement a quality management system (QMS) towards the accreditation of laboratories in the region with emphasis on the quality of point-of-care (POC) testing, biosafety and biosecurity, equipment maintenance, sample referral systems and antimicrobial resistance surveillance. The final objective was to support selected member states to develop the laboratory components of regional proposals to address antimicrobial resistance.

This article discusses the activities in the first year of the project implementation and the role of the implementation in the fight against COVID-19, which is currently the greatest global challenge.

## Description of the intervention

### Ethical considerations

This article followed all ethical standards for research without direct contact with human or animal subjects.

### Development of a framework and statute for the RISLNET

Two workshops were organised in July 2018 and March 2019 to accomplish this task. The first workshop brought countries together within the circumscription of the Central Africa Regional Collaboration Centre of Africa CDC, and this was a key step towards setting up a network of health institutes and laboratories within the region. In this forum, experts from the human, animal and environmental health sectors of the nine countries came together in a workshop in Brazzaville, Gabon, and began to share contacts, create linkages and share information and experiences within the region. The workshop, which ended with the launching of RISLNET for the Central Africa region under the patronage of the Minister of Health of the Republic of Congo, also saw the inauguration of bureau members for the Central Africa RISLNET. A second workshop was organised in Malabo, Equatorial Guinea, to guide the development of the National Laboratory Strategic Plan and Policies for member states.

To foster the development of a framework and statute for the Central Africa RISLNET, frameworks and guidelines were developed that included: a framework and statute for RISLNET, a sample transport system framework for the Central Africa RISLNET, a quality manual template for laboratory testing, and guidelines for laboratory biosafety and biosecurity.

A website was also developed for the Central Africa RISLNET to enhance the sharing of public health information, laboratory quality best practices and laboratory quality documents, as well as to encourage networking among public health laboratories in the Central Africa region.

### Mapping and linkage of centres of excellence and laboratories in the region using the Extension for Community Health Outcomes platform

The Extension for Community Health Outcomes (ECHO) was launched at GHSS’ head office in Douala, Cameroon, to support the mentorship and supervision of seven laboratories enrolled for accreditation. These laboratories were linked and coordinated together with centres of excellence and public health laboratories for event-based and laboratory-based disease surveillance. They include Laboratoire l’hôpital General de Reference National, N’Djamena, Chad; Laboratoire de L’hôpital Prince Regent Charles, Bujumbura, Burundi; Laboratoire National de Sante Publique, Brazzaville, Congo Brazzaville; Laboratorio National de Referencia Para Tuberculose Sao Tome, Sao Tome and Principe; International Centre of Medical Research of Franceville, Franceville, Gabon; Laboratorio Castroverde, Malabo, Equatorial Guinea; and Laboratoire National de Biologie Clinique et de Sante Publique, Bangui, Central African Republic. The sample transport and information sharing systems between these laboratories and countries’ centres of excellence and national reference laboratories have been linked to ECHO.

### Implementation of QMS and biosafety in the region

A training workshop was held in N’Djamena, Chad, from 22 July 2019 to 26 July 2019. Biologists, laboratory managers, and laboratory technicians were trained on laboratory QMS and the implementation of immediate improvement projects to speed up the World Health Organization’s Regional Office for Africa accreditation process. The training served as a springboard for International Organization for Standardization-15189 accreditation, as 21 participants were trained on productivity management, quality assurance, documents and records management, and the use of the Strengthening Laboratory Management Towards Accreditation (SLMTA) toolbox.

### Development of a framework for implementing QMS for POC testing

This activity was designed to increase the accuracy and reliability of POC testing, which is useful for the rapid detection of endemic and outbreak diseases. A 5-day training of trainers was conducted from 28 October 2019 to 01 November 2019 in Libreville, Gabon, to build the capacity of 26 healthcare personnel to support the institutionalisation and sustainability of POC testing. This activity aimed to ensure that quality assurance officers are well equipped with the skills, knowledge and abilities necessary to perform assigned tasks. The World Health Organization/CDC-approved POC comprehensive quality assurance training package was used, with emphasis on the Stepwise Process for Improving the quality of HIV-Related Point-of-Care Testing checklist.^9^

### Implementation of the Computing for Good (C4G) BLIS

Eight hospital laboratories, one public health laboratory and one reference laboratory each in Burundi, Congo, Chad, and Sao Tome and Principe, benefited from the Computing for Good (C4G) Basic Laboratory Information Systems (BLIS) network.

After a baseline survey, gaps identified in each of the laboratories were addressed to allow the implementation of the BLIS network, and the GHSS team embarked on the implementation phase to install and implement a C4G BLIS network in these laboratories to improve turn-around times and to support clinical decision making. The GHSS team also purchased and installed information technology materials and network connectivity to support the management of C4G BLIS in the laboratories. The laboratory staff were then trained on the use of BLIS for the proper management of laboratory data and the day-to-day activities of the laboratory.

## Lessons learnt

The COVID-19 disease is entirely new, and the characteristics of its causative virus, the severe acute respiratory syndrome coronavirus-2, are not fully understood.^10^ Drug development typically takes years of scientific studies, and unfortunately, the new coronavirus has not given the world that length of time. At the start of the pandemic, there was no defined guideline available for the management of the disease.

The ECHO platform provides practitioners with the opportunity to come together weekly to share experiences from the treatment centres in different countries. This will enhance patient management as expertise on better patient management strategies will easily be shared among different countries. This platform will also permit various treatment centres to discuss treatment challenges and receive feedback and contributions from colleagues.

There is a great need to boost fragile health systems like those in the Central Africa region for better disease surveillance and outbreak response. A body such as GHSS with vast experience in supporting health systems in Central Africa can utilise the ECHO platform established in the region to support the respective countries as they respond to this pandemic. It can guide infection prevention and control and virtual training on biosafety practices and sample collection, packaging and transportation for healthcare workers via the ECHO platform, all of which are crucial in the fight against COVID-19. This platform is now used regularly by GHSS to strengthen the quality of testing in Cameroon and the Democratic Republic of Congo.

The RISLNET initiative, after one year of its implementation, has been very helpful in the fight against the COVID-19 outbreak. At a moment when health systems across the globe are facing significant challenges, it is the product of the RISLNET initiative (the elected centres of excellence, referral network, trained personnel, and developed guidance documents) that has formed the basis of the sub-region’s strategy in the fight against COVID-19. As COVID-19 testing is not entirely decentralised within member states, the sample transport system framework for RISLNET Central Africa can easily be adapted to the COVID-19 sample transport network within member states. Furthermore, the laboratory testing quality manual template put in place will guide good laboratory practice for COVID-19 testing and ensure timely, accurate and reliable results. The guidelines for laboratory biosafety and biosecurity should provide an appropriate foundation for the implementation of safety practices for the protection of frontline healthcare workers who are most at risk.

Most importantly, the RISLNET website^11^ has been essential in the sharing of information of public health interest, laboratory quality best practices, and laboratory quality documents. The website contains the emergency numbers of member states, provides updates on the COVID-19 situation and enhances the sharing of COVID-19 strategic documents, recommendations, guidelines, feedback from meetings, etc.

The SLMTA programme was established because the United States CDC, World Health Organization’s Regional Office for Africa and stakeholders recognised the poor state of medical laboratory systems in Africa and prioritised the need to strengthen them to fight multiple diseases.^4^ Few laboratories in Central Africa recognise the importance of developing QMSs, laboratory strategic plans, and protocols that facilitate continuous laboratory quality improvement for patient care. The implementation of SLMTA in Africa has improved laboratory quality, biosafety, standardisation, maintenance, record keeping, and reporting in Africa, all of which are crucial to the optimal function of laboratories.^12,13^ The laboratories that participated in the RISLNET initiative were mentored for accreditation using the SLMTA toolkit. It is therefore not surprising that these laboratories now serve as reference laboratories for COVID-19 testing in their respective countries. The countries would have found it challenging to quickly respond to the COVID-19 outbreak if the laboratories had not received training on QMS, quality assurance, biosafety and biosecurity, and equipment maintenance and calibration.

Also, POC testing has been at the centre of the strategy to scale-up COVID-19 testing in Central Africa. Notably, GeneXpert, which is currently being used in scaling up COVID-19 testing in Central Africa, was the focus of this implementation. The participants trained in the implementation of QMS for POC testing are now adequately equipped to lead their countries in the scale-up of COVID-19 testing. Participants were also trained on capacity development for healthcare personnel to support the institutionalisation and sustainability of POC testing. Thus, they can easily initiate, implement, evaluate and provide corrective action to the various POC sites carrying out COVID-19 diagnoses.

The use of a C4G BLIS network in these laboratories will improve turn-around times for COVID-19 testing, which is very critical for clinical decision making, surveillance efforts, and the enforcement of preventive measures. The provision of information technology materials and network connectivity to support the management of C4G BLIS will facilitate data sharing and communication between the testing laboratories and collection sites. Laboratory staff trained on the proper use of BLIS can properly manage testing data and rapidly generate and report testing statistics to health authorities for quick decision making.

The impact of these project implementation activities on the regional response to COVID-19 cannot be understated but may be difficult to quantify. Nonetheless, lessons learned in the past have shown that strengthening laboratory systems is the best strategy for outbreak preparedness and response. During the Ebola epidemic in West Africa between 2013 and 2016, laboratory systems were overwhelmed, and the medical world saw the need to rethink the future of global laboratory medicine. In West Africa, the laboratory system could not diagnose haemorrhagic fevers and other diseases and, as a result, the public health experts failed to notice the outbreak in time.^5^ Resultant from weak laboratory systems, public health authorities in the West Africa region continues to miss Ebola cases, leading to Ebola illnesses and deaths erroneously attributed to other infections.^14^ Similarly, the World Health Organization was only notified of the Ebola outbreak characterised by fever, severe diarrhoea, vomiting, and a high fatality rate in Guinea in March 2014; however, epidemiological investigations linked laboratory-confirmed cases with a presumed first fatal case of the outbreak in December 2013.^15,16^ Thus, weak laboratory systems frustrate surveillance systems and decrease the quality of patient care, which are integral parts of the preventive and therapeutic interventions needed in disease control.

## Recommendations

Expanding SLMTA to other laboratories within member states in the Central Africa region will significantly improve the quality management in these laboratories and result in a better healthcare system. The laboratories enrolled in this programme can be further supported through supportive site visits or coaching through the ECHO platform; this will improve their effectiveness in responding to COVID-19 testing challenges. Outlining a clear plan to support these sites will help sustain efforts towards achieving accreditation and boost their current role in the fight against COVID-19. More funding is required for these efforts and the improvement of health systems in Central Africa.
